# Robotic complete mesocolic excision using indocyanine fluorescence imaging in colorectal cancer: A case study and technical approach

**DOI:** 10.1016/j.ijscr.2020.01.038

**Published:** 2020-02-06

**Authors:** R. Young, A.K.S. Rajkomar, P. Smart, S.K. Warrier

**Affiliations:** a Department of Surgery, Melbourne Health, Melbourne, Victoria, Australia; b Gastrointestinal Clinical Institute, Epworth Healthcare, Victoria, Australia; c Department of Surgery, Austin Health, Victoria, Australia; d Department of Cancer Surgery, Peter MacCallum Cancer Centre, Victoria, Australia

**Keywords:** Case report, Robotic, Complete mesocolic excision

## Abstract

**Introduction:**

Colorectal cancer is the second most common malignancy in developed countries and accurate staging is vital for determining the most appropriate management plan, in particular, whether adjuvant chemotherapy is recommended in addition to surgical resection. There is currently no consensus regarding standard practice for lymph node resection in colorectal cancer although there is increasing evidence to support the use of sentinel lymph node mapping to target lymph node sampling and facilitate ultra-staging of nodes.

**Presentation of case:**

We present the case of a 49 year old female who underwent a robotic right hemicolectomy and complete mesocolic excision for caecal adenocarcinoma using indocyanine fluorescence imaging (ICG FI) to guide nodal dissection.

**Discussion:**

ICG FI may be useful for sentinel lymph node mapping in colorectal cancer. This technique can assist to identify the first draining lymph nodes and permit ultra-staging of lymph nodes.

**Conclusion:**

While there is still limited evidence to support ICG FI, the current body of literature suggests that it is likely to be a feasible and sensitive technique for guiding sentinel lymph node sampling in colorectal cancer.

## Highlights

• Complete mesocolic excision confers a possible survival advantage in right sided colorectal cancer.
• Indocynanine Green (ICG) Fluorescence Imaging may allow for more targeted lymph node clearances.
• Robotic surgery allows for these nodes to be removed with an MIS approach.

## 1 Introduction

Colorectal cancer is the second most common malignancy in developed societies and is responsible for the second largest number of cancer-related deaths [1]. Accurate staging of colorectal cancer is vital as this the overall management approach, in particular, whether or not adjuvant chemotherapy is recommended in addition to surgical resection. Adjuvant chemotherapy is generally recommended for Stage III (node positive) colorectal cancer and thus thorough lymph node staging is of utmost importance [1]. Around 30% of patients with colorectal cancer are under-staged and will go on to develop recurrent nodal disease despite being initially deemed node negative on histopathological analysis [2]. Under-staging may be due to inadequate lymph node sampling or failure to diagnose micro-metastatic disease which can be difficult to identify on histopathological analysis alone. Ultra-staging (serial sectioning and use of immunohistochemistry) may assist with the identification of micro-metastatic nodal disease, however, these techniques are time consuming and costly to undertake on all sampled lymph nodes [3]. Identification of sentinel lymph nodes in colorectal cancer may be beneficial in terms of guiding lymph node dissection and permitting more in analysis of high yield nodes. Several techniques for identifying sentinel nodes and undertaking nodal dissection have been described in the literature. There is mounting evidence to support the use of Indocyanine Fluorescence Imaging (ICG FI) to guide nodal dissection in colorectal cancer [3,4].

This work has been reported in line with the SCARE criteria [5].

## 2 Presentation of case

A 49-year-old female who underwent robotic right hemicolectomy and complete mesocolic excision (CME) for caecal adenocarcinoma using Indocyanine Fluorescence Imaging to guide nodal dissection.

The patient presented with iron-deficiency anaemia. She underwent gastroscopy and colonoscopy and was found to have a clearly malignant lesion at the ileocaecal valve in addition to multiple colonic polyps. Histopathology from two descending colon polyps returned showing tubulovillous adenoma with high grade dysplasia and tubular adenoma with low grade dysplasia respectively. The malignant lesion was not biopsied pre-operatively. Pre-operative staging using CT and PET scanning and did not demonstrate metastatic disease. She presented for surgical resection.

## 3 Technical approach

**Fig. 1:**
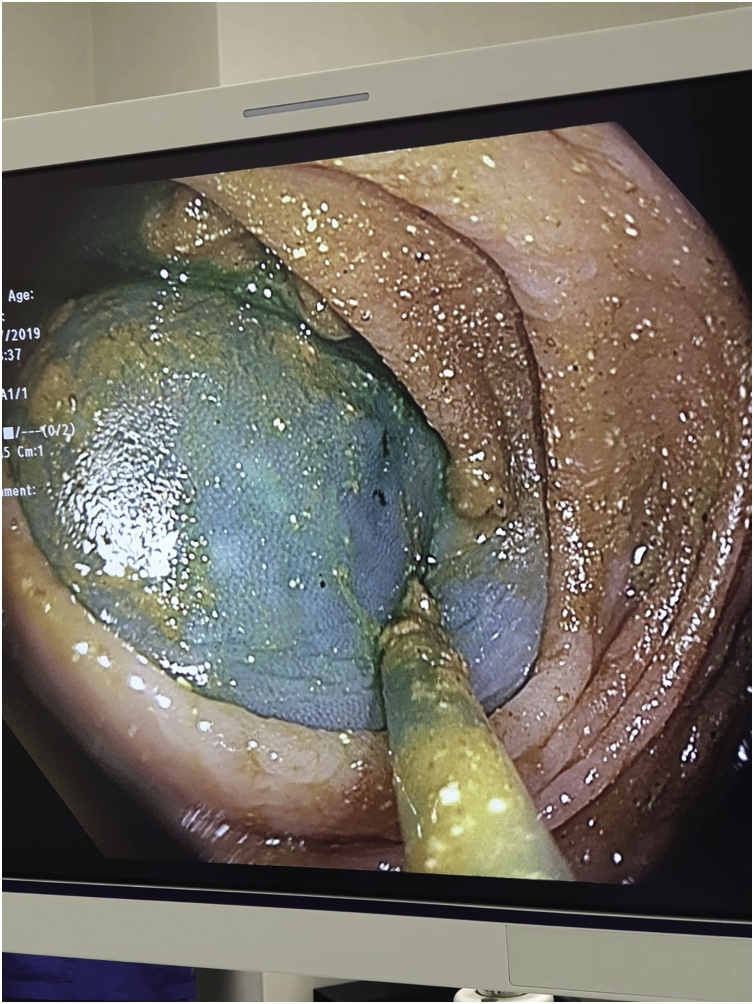
Endoscopic photograph demonstrating injection of Indocyanine Fluorescence dye.

**Fig. 2:**
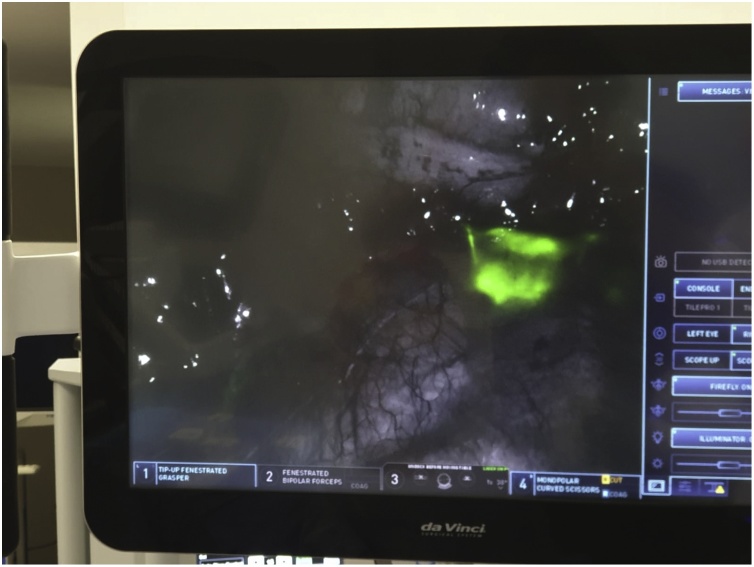
Intra-operative photograph demonstrating fluorescence in lymph nodes.

The patient was positioned supine with a standard four port robotic Xi technique employed. Prior to commencing surgical resection, colonoscopy was repeated and again demonstrated the presence of multiple colonic polyps including several 10–12 mm polyps in the rectum and at the rectosigmoid junction. These polyps were hot-snared and excised and sent for histopathological examination along with the final surgical specimen. Indocyanine (ICG) injection was then introduced via colonoscopic guidance to assist with sentinel lymph node mapping (see Fig. 1, Fig. 2).

The da Vinci® System Firefly fluorescence imaging (Surgical Intuitive, Sunnyvale, United States of America) subsequently demonstrated fluorescence in the lymph nodes surrounding the middle colic vessels and complete mesocolic excision was undertaken. For the CME dissection, robotic targeting was aimed at the hepatic flexure and arm 3 was used as the camera port. Arms 1 and 2 were used as left-handed retracting instruments while arm 4 was used as the main dissector (advanced bipolar energy or monopolar cautery with scissors). A superior mesenteric vein (SMV) first technique was adopted for the CME dissection whereby all lymphatic tissue overlying the SMV was removed. The dissection was continued to the right branches of the middle colic vessels which were dissected and divided. Upon completion of the vascular dissection, the remainder of the anatomical dissection was completed. This was performed with a medial to lateral approach that used embryological planes and respected the underlying retroperitoneal structures. A standard robotic ileocolic intracorporeal anastomosis was performed with a 60 mm blue load Sureform®™ stapler and 3-0 V-Loc™ to close the common enterotomy in a single layer.

Final histopathology from the surgical resection demonstrated a T3N1b moderately differentiated adenocarcinoma with two nodes involved out of a total of 47 excised nodes. There were multiple adenomatous polyps in the right hemicolectomy specimen as well as the excised rectosigmoid polyp which were found to be tubulovillous adenomata. Molecular pathology was undertaken on the primary colonic tumour and the lesion was found to have a KRAS mutation (G12C mutation and a PTEN mutation (D107Y and H141D mutations). Due to the presence of involved lymph nodes, the patient was treated with adjuvant FOLFOX chemotherapy.

## 4 Discussion

Adequate lymph node sampling is critical for correctly staging colorectal cancers. There is currently no standard recommendation for lymph node resection in colorectal cancer, however, there is increasing discussion about the use of sentinel node biopsy to target lymph node resection and allow for ultra-staging of nodes (serial sectioning and use of immunohistochemistry) harbouring micro-metastatic disease [3]. Techniques for complete mesocolic lymph node excision (CME) have been described in the literature, with research suggesting that CME may increase the yield of lymph nodes and improve overall survival in patients with primary colorectal cancers [6,7]. In recent times, research efforts have been directed toward investigating the use of fluorescence imaging to improve targeting of lymph node resection in CME.

Indocyanine green fluorescence imaging (ICG FI) is increasingly being used to improve accuracy of staging in primary colorectal cancer patients by assisting in the identification and sampling of lymph nodes in CME. The development of ICG FI stems from the understanding of sentinel lymph node in breast cancer and melanoma (i.e. the first lymph node draining a cancer) [8,9]. Sentinel lymph node sampling traditionally involves injecting a coloured dye or radioactive marker into peri-tumoural tissue which then spreads to involve the first draining lymph nodes. Use of ICG involves the same technique using a fluorescent marker which can be identified in involved lymph nodes by infra-red scanning. One advantage of this technique is that it allows for real time lymphography. The evidence for ICG FI in colorectal cancer is limited at present, but the current body of literature suggests overall feasibility and sensitivity of this technique in detecting involved nodes [3].

## 5 Conclusion

ICG FI is increasingly being utilised to target lymph node resection in colorectal cancer. While there is still limited evidence to support ICG FI, the current body of literature suggests that it is likely to be a feasible and sensitive technique for guiding sentinel lymph node sampling in colorectal cancer.

## Declaration of Competing Interest

Nil to declare.

## Funding

This study was supported by Epworth Research Institute Major Research Grant No. 11.952.000.80982.

## Ethical approval

This study has been exempt from ethical approval at our institution, however, permission has been sought from the patient in question to present and publish this case report.

## Consent

Consent has been obtained (written and signed) from the patient in question to complete this case report.

## Author’s contribution

Dr Rebekah Young: Conceptualisation, writing of original draft.

Mr Amrish Rajkomar: manuscript editing.

Mr Phil Smart: Data curation, conceptualisation, funding acquisition, review and editing of manuscript.

Mr Satish Warrier: Data curation, conceptualisation, funding acquisition, review and editing of manuscript.

## Registration of research studies

Not applicable – case report only.

## Guarantor

Mr Phillip Smart.

## Provenance and peer review

Not commissioned, externally peer-reviewed.
